# Adina Rubella‐Like Microsized SiO@N‐Doped Carbon Grafted with N‐Doped Carbon Nanotubes as Anodes for High‐Performance Lithium Storage

**DOI:** 10.1002/smsc.202100105

**Published:** 2022-01-12

**Authors:** Weilan Xu, Cheng Tang, Na Huang, Aijun Du, Minghong Wu, Jiujun Zhang, Haijiao Zhang

**Affiliations:** ^1^ Institute of Nanochemistry and Nanobiology Shanghai University Shanghai 200444 China; ^2^ School of Chemistry Physics and Mechanical Engineering Science and Engineering Faculty Queensland University of Technology Brisbane QLD 4001 Australia; ^3^ School of Environmental and Chemical Engineering Shanghai University Shanghai 200444 China; ^4^ Institute for Sustainable Energy College of Sciences Shanghai University Shanghai 200444 China

**Keywords:** carbon nanotubes, in situ catalytic growth, lithium-ion batteries, micrometer-sized SiO, nitrogen doping

## Abstract

Microsized silicon oxide (SiO) has become a highly potential anode material for practical lithium‐ion batteries (LIBs) in virtue of its low cost and high capacity. However, its commercialization is still impeded by the low inherent conductivity and nonignorable volume expansion of SiO in the lithiation/delithiation processes. Herein, an in situ catalytic growth approach is developed for grafting N‐doped bamboo‐like carbon nanotubes (NCNTs) onto the polydopamine‐coated SiO microparticles, yielding a unique adina rubella‐like SiO@NC‐NCNT composite. The cross‐sectional scanning electron microscopy images reveal that the flexible middle‐carbon layer plays a crucial role in alleviating volume expansions and improving structural stability of SiO@NC‐NCNTs. Theoretical density functional theory simulation results further prove that the rational construction of ternary heterostructure can effectively balance lithium adsorption energies and greatly improve conductivity of SiO@NC‐NCNTs. As a result, the as‐fabricated SiO@NC‐NCNTs LIB anode shows a high reversible specific capacity of 1103.7 mA h g^−1^ at 0.2 A g^−1^ after 200 cycles with a high retention of 99.6% and an outstanding rate capability of 569 mA h g^−1^ at 5000 mA g^−1^. The strategy developed herein demonstrates a feasible avenue for developing high‐energy SiO‐based anodes for LIBs.

## Graphical abstract

An in situ catalytic growth method is developed for grafting N‐doped bamboo‐like carbon nanotubes (CNTs) onto the carbon‐coated SiO microparticles, forming unique adina rubella‐like SiO@NC‐NCNT composite. With these structural and compositional advantages, the resulting SiO@NC‐NCNT anode demonstrates excellent lithium storage performance.

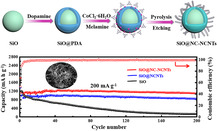

## 1 Introduction

With the extensive exploration of clean and sustainable energy sources such as solar, wind, waterfall, geothermal, etc., the rapid development of electrochemical energy storage and conversion technologies for the electricity generated from such intermittent sources has been seen in most recent years. Among different electrochemical energy storage and conversion technologies, lithium‐ion batteries (LIBs) have become one type of the most reliable, efficient, and practical devices for many applications such as electric vehicles, portable powers, and stationary power plants due to their high energy/power densities, relatively long cycle life, and friendly environmental compatibility.^[^ ^1^, ^2^ ^]^ In the effort to further increase the energy density to prolong the recharge mileage of the devices, besides increasing the capacity and voltage of cathode materials, developing high‐capacity anode materials has been identified as an effective way.^[^ ^3^ ^]^ However, the current commercial graphite anodes are difficult to meet the demand of high energy density of the batteries because of their low theoretical specific capacities.^[^ ^4^ ^]^ Therefore, it is necessary to develop ideal anode materials for practical LIBs with low cost, high capacity, and good durability.

Silicon (Si)‐based materials have long been considered to be one type of the most potential anodes for next‐generation high‐energy LIBs owing to their high theoretical capacity (4200 mA h g^−1^), low delithiation potential (<0.5 V), rich reserves, and low cost.^[^ ^5^, ^6^, ^7^ ^]^ However, Si‐based anodes suffer from drawbacks including huge volume change (≈400%) during charge/discharge and poor internal conductivity. Although Si‐based nanomaterials with small particle sizes can improve the transmission dynamics of electrons and Li ions, their high cost seriously hinders the large‐scale commercial applications. Alternatively, microsized Si‐based materials have attracted attention because of their high tap densities and low preparation cost. For example, silicon monoxide (SiO) with much less volume expansion has emerged as a promising substitute for Si recently.^[^ ^8^ ^]^ However, its poor ionic/electronic conductivities are not conductive to the transport of Li ions and electrons, and its volume change in long‐term cycles is still seen an issue. Especially, the microsized SiO also has polarization of particle crushing.^[^ ^9^, ^10^ ^]^

To tackle the earlier issues, the composite microsized SiO with conductive carbon material such as graphene, amorphous carbon, or carbon nanotubes (CNTs) could provide the increased electronic conductivity and effectively relieve mechanical stress during deep cycles, thereby improving the electrochemical performances of these material‐based anodes for LIBs.^[^ ^11^, ^12^, ^13^, ^14^ ^]^ Among differently composed carbon materials,1D CNTs can not only shorten the transmission path of ions and electrons, but also easily form a stable 3D conductive network for the microsized SiO composite anodes.^[^ ^15^, ^16^, ^17^ ^]^ For example, Guo and co‐workers polymerized the partially LiOH‐neutralized acrylic acid (Li‐AA) on carbon‐coated SiO_*x*_ microparticles and then uniformly interfused CNTs on the Li polyacrylate interface to enhance the close contact with carbon coating for improving the Li storage capability.^[^ ^8^ ^]^ It seemed that maintaining structural integrity could be a challenge due to the weak binding between CNTs and carbon‐coated SiO_*x*_ induced by the huge volume effect. Li and co‐workers prepared Si@N‐doped CNTs by pyrolysis of the Co−zeolitic imidazole framework (ZIF‐67), and the resulting composite could effectively prevent pulverization and accommodate the volume fluctuation of Si during cycling.^[^ ^18^ ^]^ However, the synthesis process seemed relatively complex and of high cost. Therefore, it still remains a challenge to achieve a highly stable composite of SiO and CNTs through a feasible and easy operation route.

In the effort to develop high‐performance anode materials for LIBs, we have achieved a facile and scalable catalytic pyrolysis process for synthesizing core−shell particles with microsized SiO core and N‐doped carbon shell (SiO@NC) on which N‐doped CNTs (NCNTs) are in situ grown to form a composite (SiO@NC‐NCNTs). The in situ growth of NCNTs onto the core−shell SiO@NC can form a highly stable 3D conductive network, further enhancing the mechanical strength. More importantly, the flexible NC shell layer derived from polydopamine (PDA)‐tightly grafted NCNTs can effectively alleviate the large volume expansions during the cycling process, as verified by the cross‐sectional scanning electron microscopy (SEM) images. Meanwhile, the double N doping can also improve the wettability and conductivity of the anode material and provide more additional lithium storage sites. Accordingly, when this SiO@NC‐NCNT is used as the anode for LIBs, both higher reversible capacity and better cycle durability in comparison with SiO@NCNTs and pristine SiO electrodes are achieved. The enhancement mechanism for the electrochemical performance is also investigated by the theoretical calculations for fundamental understanding.

## 2 Results and Discussion

### 2.1 Material Synthesis and Characterizations

**Figure 1:**
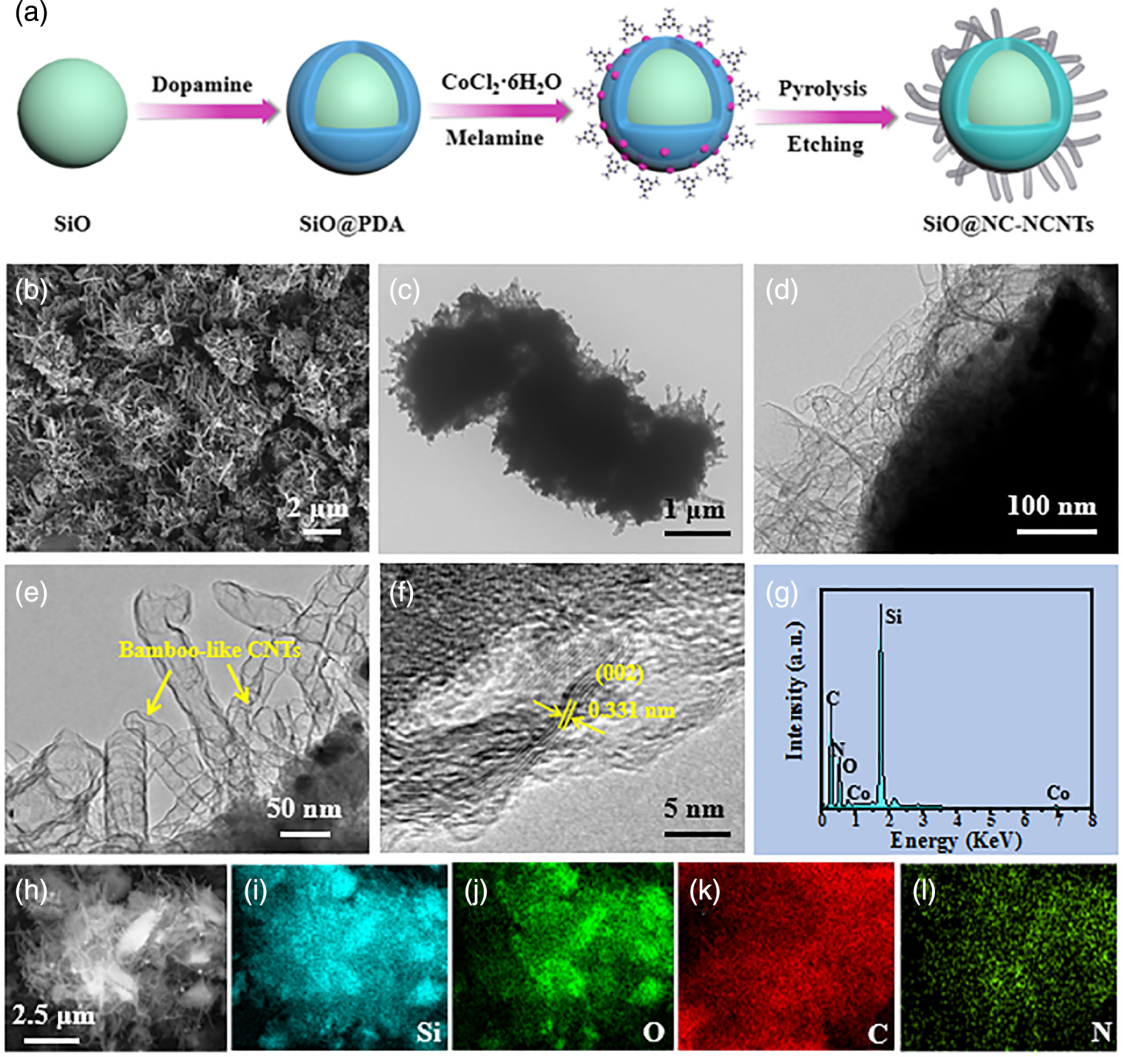
a) Schematic illustration of the formation process of microsized SiO@NC‐NCNTs composite. b) SEM image, c–e) TEM images, f) HRTEM image, g) EDS spectrum, h) STEM image of SiO@NC‐NCNTs, and i–l) the corresponding elemental mapping of Si, O, C, and N.

**Figure** **1**a describes the fabrication procedures for microsized SiO@NC‐NCNT particles. First, a thin PDA layer of about 20 nm is uniformly coated onto the surface of commercial SiO with a particle size of 2–3 μm (Figure S1, Supporting Information), generating a core−shell SiO@PDA structure (Figure S2, Supporting Information). After that, the as‐prepared SiO@PDA particles are mixed with CoCl_2_ and melamine, which are used as the catalyst and the carbon precursor, respectively. In this regard, PDA with rich hydroxyl groups can attract positively charged Co^2+^ and melamine via electrostatic interactions, resulting in a uniform distribution of Co^2+^ and melamine onto the SiO surface. Subsequently, the obtained precursor is placed into a tubular furnace and heated at 800 °C in flowing N_2_ atmosphere, where the PDA layer is converted into the N‐doped carbon layer, and melamine is pyrolyzed into a gaseous carbon source and adsorbs on the surface of Co catalyst. Following the Co‐catalyzed tip‐growth mechanism, the bamboo‐like NCNTs are in situ grafted onto the surface of the NC layer.^[^ ^19^ ^]^ Finally, the optimized SiO@NC‐NCNT product is successfully obtained after removing the formed Co nanoparticles via an acid‐etching process.

Electronic microscopy techniques were conducted to investigate the microscopic morphology and the detailed structure of the products. Figure 1b shows the SEM image of SiO@NC‐NCNTs, where a lot of vertical CNTs are homogeneously grown onto the surface of SiO@NC, showing a well‐defined morphology. Interestingly, SiO@NC‐NCNTs show a similar morphology to natural adina rubella. Transmission electron microscopy (TEM) images further confirm that NCNTs are firmly connected with SiO@NC (Figure 1c–e), and the average diameter of NCNTs is 35 nm. Particularly, NCNTs show a distinct bamboo‐like structure, as seen from the magnified TEM image in Figure 1e, which should be in favor of improving the electrical conductivity.^[^ ^20^ ^]^ The controlled experiments indicate that the SiO@NCNT sample has also a similar morphology to SiO@NC‐NCNTs except for the middle carbon layer (Figure S3, Supporting Information), probably due to the presence of hydroxyl groups on the SiO surface. Importantly, without the Co catalytic effect, only some irregular carbon nanosheets can be found around SiO@NC (Figure S4, Supporting Information), indicating the key role of Co catalyst. Figure 1f shows the high‐resolution transmission electron microscopy (HRTEM) image of SiO@NC‐NCNTs. It can be seen that the lattice spacing is 0.331 nm, which is in line with the (002) crystal plane of graphic carbon.^[^ ^21^ ^]^ The EDS result (Figure 1g and S5, Supporting Information) demonstrates the coexistence of Si, O, C, N, and Co elements. The Scanning transmission electron microscope (STEM) image and the corresponding elemental mapping verify that Si, O, C, and N elements are homogeneously distributed over the whole SiO@NC‐NCNTs sample (Figure 1h–l), suggesting the good combination of the middle NC layer and NCNTs with SiO particles.

**Figure 2:**
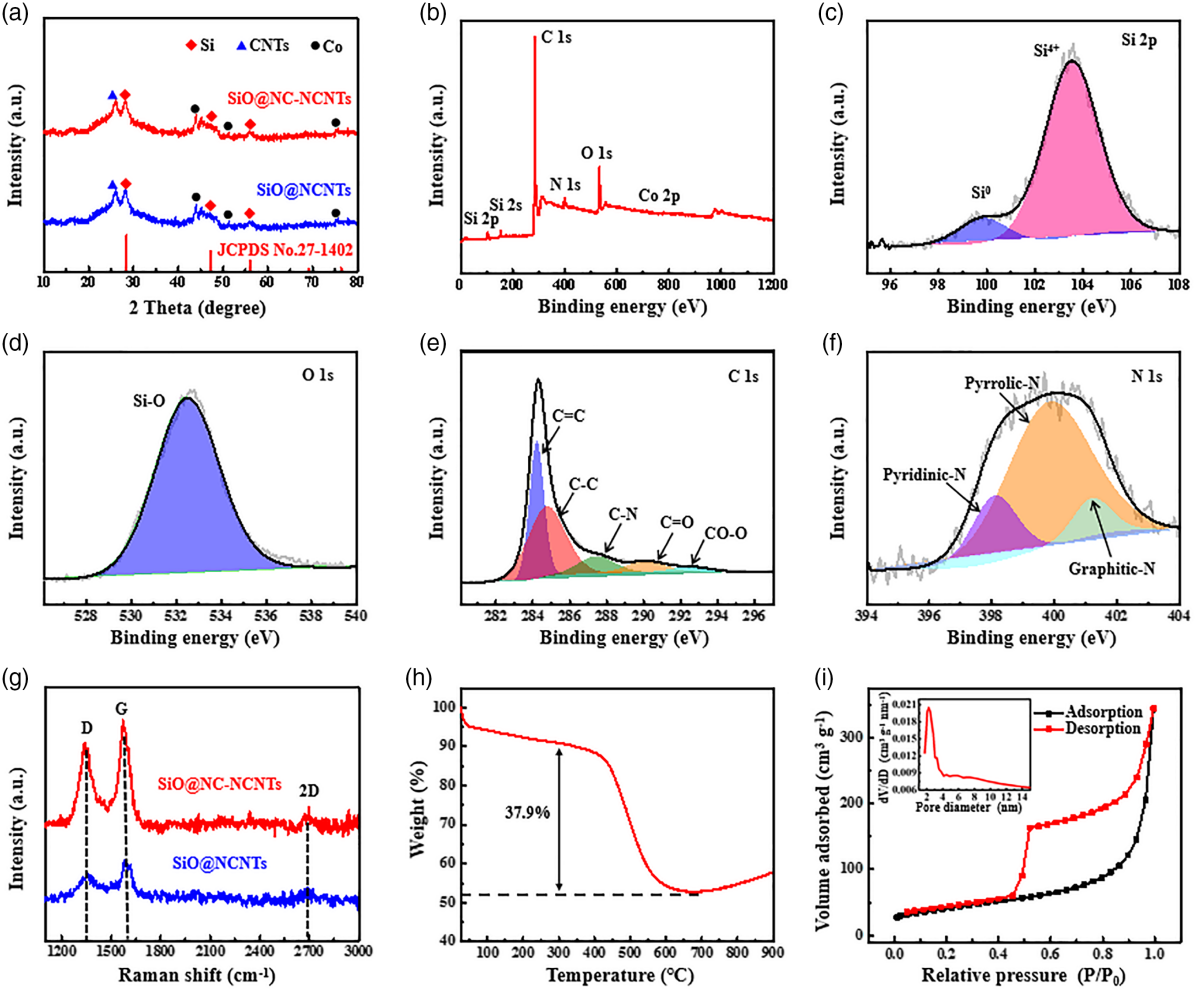
a) XRD patterns of SiO@NC‐NCNTs and SiO@NCNTs. b) XPS survey spectrum and high‐resolution XPS spectra of c) Si 2*p*, d) O 1*s*, e) C 1*s*, and f) N 1*s* of SiO@NC‐NCNTs. g) Raman spectra of SiO@NC‐NCNTs and SiO@NCNTs. h) TGA curve and i) N_2_ adsorption−desorption isotherm and the corresponding pore size distribution (inset) of SiO@NC‐NCNTs.

**Figure** **2**a shows the X‐ray diffraction (XRD) patterns of SiO@NC‐NCNTs and SiO@NCNTs. The diffraction peaks at 28.4°, 47.3°, and 56.1° are assigned to (111), (220), and (311) crystal planes of Si (JCPDS No. 27‐1402), respectively, which is different from the pristine SiO with only a broad characteristic peak of amorphous SiO_2_ (Figure S6, Supporting Information). The peak at 26.3° corresponds to the (002) crystal plane of graphite carbon (JCPDS No. 41‐1487), confirming the CNT formation. The peaks of metal Co (JCPDS No. 15‐0806) at 44.2°, 51.4°, and 75.8° mainly result from the residual Co particles after etching.

The chemical states and surface composition of SiO@NC‐NCNTs were investigated using X‐ray photoelectron spectra (XPS). The XPS full survey spectrum in Figure 2b reveals the presence of Si, C, N, and O in the sample, while the Co 2*p* peak is almost invisible, further indicating that most Co nanoparticles have been removed. Figure 2c shows the high‐resolution Si 2*p* spectrum, and two peaks at 99.8 and 103.5 eV are ascribed to Si^0^ and Si^4+^, in line with the XPS results of pristine SiO (Figure S7, Supporting Information). Meanwhile, the O 1*s* high‐resolution spectrum (Figure 2d) shows the peak of Si–O (532.4 eV).^[^ ^22^ ^]^ In Figure 2e, the C 1*s* XPS spectrum can be divided into five peaks at 284.27, 284.8, 287.4, 290.1, and 292.6 eV, which correspond to C=C, C—C, C—N, C=O, and CO—O, respectively.^[^ ^23^ ^]^ Particularly, the generation of C—N bond means that N atoms have been in situ doped into the carbon matrix.^[^ ^24^ ^]^ The N 1*s* peak from Figure 2f then affirms this fact, which consists of pyridinic N, pyrrolic N, and graphite N. The existence of C—N bond and nitrogen doping can provide more active sites for Li^+^ adsorption and regulate the surface energy barrier of the carbon substrate, resulting in the increased electrochemical performances.^[^ ^25^ ^]^

Figure 2g shows Raman spectra of two samples. The characteristic peaks at around 1343, 1571, and 2690 cm^−1^ are generally ascribed to the D, G, and 2D bands of CNTs, respectively.^[^ ^26^ ^]^ The relative intensity ratio of the peaks (*I*_D_/*I*_G_) is used to discern the graphitization degree of carbon materials.^[^ ^27^ ^]^ The *I*_D_/*I*_G_ ratio of SiO@NC‐NCNTs and SiO@NCNTs is calculated to be 0.90 and 0.84, indicating good crystallinity of NCNTs. The *I*_D_/*I*_G_ value of the sample synthesized without Co catalyst is about 0.95 (Figure S8, Supporting Information), further confirming the catalytic role of Co. TG analysis was conducted to determine the carbon contents in the composite (Figure 2h and S9, Supporting Information). The significant weight loss between 300 and 650 °C is mainly ascribed to the combustion of carbon. In addition, the curve shows the increased weight profile after 650 °C, which is due to the oxidation of SiO. By further analysis, the carbon content in SiO@NC‐NCNTs and SiO@NCNTs was estimated to be 37.9 and 29.6 wt%, respectively.

The specific surface area and pore structure were also analyzed according to N_2_ sorption isotherms (Figure 2i and S10, Supporting Information). As shown in Figure 2i, SiO@NC‐NCNTs exhibit a type‐IV isotherm with sharp capillary condensation at high relative pressure. The result indicates the existence of mesopores, which is further confirmed by the pore size distribution curve (inset in Figure 2i) with a narrow pore distribution centered on 2.3 nm. The SiO@NC‐NCNT product has a large surface area of 143.2 m^2^ g^−1^, much higher than 56.2 m^2^ g^−1^ of SiO@NCNTs. The large surface area and narrow mesoporous structure are conducive to the sufficient contact between electrolyte and electrode material and fast electrochemical reaction dynamics during the Li storage process.^[^ ^28^, ^29^ ^]^

### 2.2 Lithium Storage Performances

**Figure 3:**
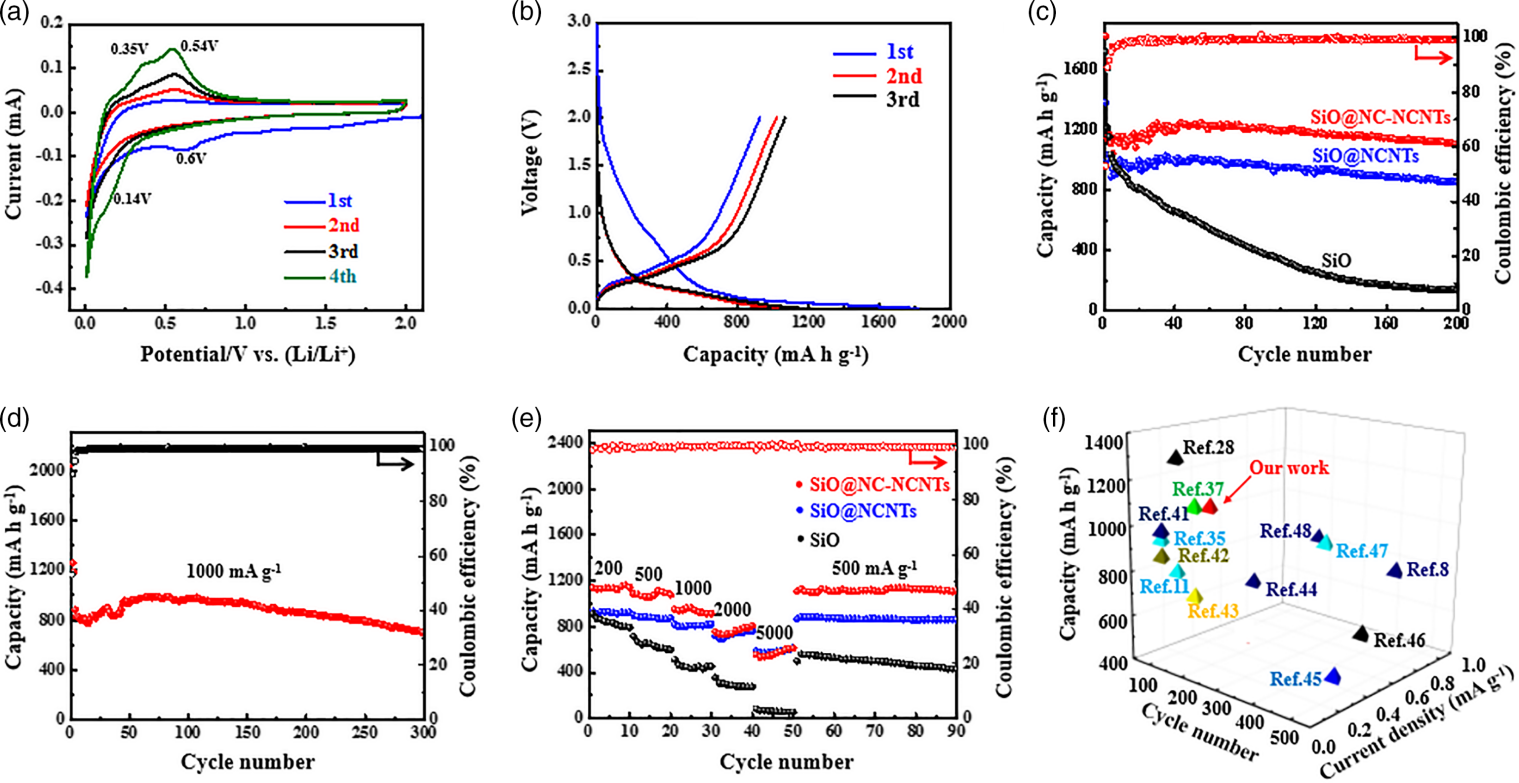
a) CV curves of the initial four cycles at a scan rate of 0.1 mV s^−1^, and b) charge−discharge profiles of the SiO@NC‐NCNTs electrode at 100 mA g^−1^. c) Cycling performances of SiO@NC‐NCNTs, SiO@NCNTs, and pristine SiO electrodes at 200 mA g^−1^ (100 mA g^−1^ for the initial three cycles). d) Cycling performance and the corresponding coulombic efficiency of the SiO@NC‐NCNT electrode at a high current density of 1 A g^−1^ (100 mA g^−1^ for the initial three cycles). e) Rate capabilities of SiO@NC‐NCNTs, SiO@NCNTs, and pristine SiO electrodes. f) Comparison of cycle performances between SiO@NC‐NCNTs and previously reported Si‐based anodes.^[^ ^41^, ^42^, ^43^, ^44^, ^45^, ^46^, ^47^, ^48^ ^]^

To evaluate the electrochemical performances of SiO@NC‐NCNTs, SiO@NCNTs, and pristine SiO anodes, the half cells were assembled with metal Li wafer as the counter electrode. **Figure** **3**a shows cyclic voltammetry (CV) curves of the SiO@NC‐NCNTs electrode between 0.001 and 2.0 V at the scan rate of 0.1 mV s^−1^ for the initial four cycles, the cathodic peak centered at 0.14 V represents the formation of Li_*x*_Si alloys,^[^ ^30^ ^]^ and a broad cathodic peak at about 0.6 V in the first cycle generally corresponds to the formation of the solid−electrolyte interface (SEI) layer and the irreversible conversion of SiO_*x*_ to active Si.^[^ ^31^ ^]^ In addition, the anodic peaks at 0.35 and 0.54 V can be ascribed to the delithiation of Li_*x*_Si phase.^[^ ^32^ ^]^ Based on the charge−discharge curves in Figure 3b and S11, Supporting Information, the initial discharge and charge specific capacity of SiO@NC‐NCNTs, SiO@NCNTs, and SiO are 1809.6 and 953.6 mA h g^−1^, 1714.4 and 1211 mA h g^−1^, and 1380.1 and 1000.2 mA h g^−1^, respectively. The initial Coulomb efficiencies (ICE) of SiO@NC‐NCNTs, SiO@NCNTs, and SiO are 53%, 63%, and 51%, and the lower ICE is mainly attributed to the relatively high surface area of SiO@NC‐NCNT, which consumes a lot of electrolytes in the early stage, and the generation of the SEI film.^[^ ^33^, ^34^ ^]^ In the third cycle, the coulomb efficiency of SiO@NC‐NCNTs increases rapidly to 91.7%, and from the fifth cycle, the coulomb efficiency is stable at above 96.7%, showing good reversibility.

The cycling durability of three electrodes was further investigated. As seen from Figure 3c, the SiO@NC‐NCNT electrode shows the best cycling stability. At the current density of 200 mA g^−1^, it delivers a high reversible capacity of 1103.7 mA h g^−1^ with a capacity retention of 99.6% (compared with the fourth cycle) after 200 cycles, much larger than those of SiO@NCNTs (851.1 mA h g^−1^) and SiO (136.9 mA h g^−1^). Even at a high current density of 1 A g^−1^ (Figure 3d), SiO@NC‐NCNTs can still give a specific reversible capacity of 700.2 mA h g^−1^ after 300 cycles, indicating durable cycling stability for fast lithium storage.

Figure 3e presents the rate capabilities of three electrodes under different current densities. At the current densities of 200, 500, 1000, 2000, and 5000 mA g^−1^, the specific discharge capacities of SiO@NC‐NCNTs are 1138.4, 1081.8, 937.6, 763, and 569.2 mA h g^−1^, corresponding to 100%, 95%, 82%, 67%, and 50% capacity retention rates, respectively. By comparison, other two electrodes have relatively lower specific capacity at each current density. When the current density returns to 500 mA g^−1^, SiO@NC‐NCNTs still have a high specific capacity of 1109 mA h g^−1^ with a 102.5% of capacity retention (compared with the capacity at 500 mA g^−1^), exhibiting an excellent electrochemical reversibility at high current density. The superior rate performance is due to the smart design of such a unique ternary heterostructure, in which NCNTs tightly grafted onto the surface of SiO@NC can withstand the large stress changes at the high current density, thus maintaining good mechanical stability for the electrode material. In addition, the cycling performance of the SiO@NC‐NCNTs electrode is also compared with those reported Si‐based anode materials, as presented in Figure 3f and Table S1, Supporting Information. Briefly, the increased lithium storage performance of SiO@NC‐NCNTs is mainly assigned to the enhanced conductivity and double protection effect of NC and NCNTs, which enable the full utilization of the energy storage capability of SiO.

### 2.3 Dynamic Analysis and Theoretical Simulations

**Figure 4:**
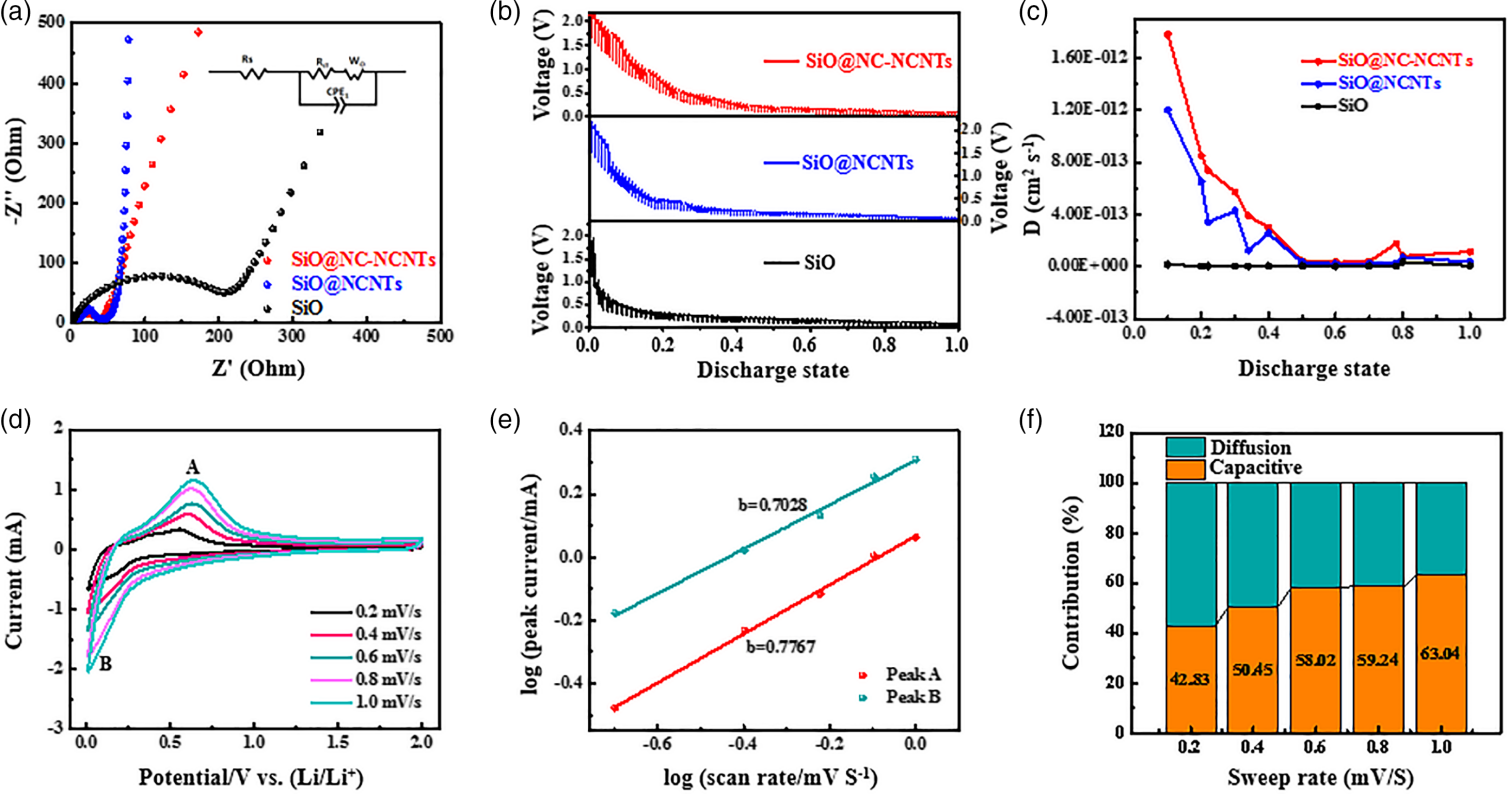
a) Electrochemical impedance spectra, b) GITT curves of SiO@NC‐NCNTs, SiO@NCNT, and pristine SiO electrodes, and c) the calculated Li chemical diffusion coefficients based on GITT results. d) CV curves of SiO@NC‐NCNTs at various scan rates of 0.2–1 mV s^−1^. e) Relationship between the logarithm peak current and logarithm sweep rate, and f) the percentages of capacitive and diffusion‐controlled capacities at different scan rates of the SiO@NC‐NCNT electrode.

Electrochemical impedance spectroscopy (EIS) was applied to probe the conductivity and ion diffusion behavior of the electrode. **Figure** **4**a displays the Nyquist plots of the freshly assembled batteries. It has been acknowledged that a semicircle in the high‐frequency region is attributed to the charge transfer resistance (*R*_ct_), while the sloping line in the low‐frequency region is related to Warburg impedance (*Z*_W_).^[^ ^35^ ^]^ The *R*_ct_ of SiO@NC‐NCNTs electrode is 42.3 Ω, which is apparently lower than those of SiO@NCNT (65.9 Ω) and SiO (228.3 Ω) electrodes. This result further indicates that the full combination of NCNTs and NC layer can significantly increase the conductivity and interfacial stability.

Galvanostatic intermittent titration technique (GITT) was performed to further study the dynamic process of electrodes (Figure 4b,c).^[^ ^16^ ^]^ The chemical diffusion coefficient of Li^+^ (*D*_Li+_) can be evaluated according to the known formula.

(1) $$D=\frac{4}{\pi \tau }{\left(\frac{n_{m}V_{m}}{S}\right)}^{2}{\left(\frac{\Delta E_{S}}{\Delta E_{\text{τ}}}\right)}^{2}$$

where *n*_m_ and *V*_m_ is the molar mass and molar volume of electron material, *τ* is the duration of the pulse, *S* is the contact area of electrode/electrolyte, and Δ*E*_S_ and Δ*E*_τ_ are the voltage changes caused by pulse and constant current charge discharge, respectively. From the results, it can be obtained that the *D*_Li+_ value of SiO@NC‐NCNTs (3.22 × 10^−14^–1.78 × 10^−12^ cm^2^ s^−1^) is higher than those of SiO@NCNT (1.84 × 10^−14^–1.20 × 10^−12^ cm^2^ s^−1^) and SiO (1.88 × 10^−16^–3.32 × 10^−14^ cm^2^ s^−1^), further confirming the fast transport dynamics of the SiO@NC‐NCNTs electrode.

To better understand the lithium storage mechanism of the SiO@NC‐NCNTs electrode, a series of CV curves were collected at different scanning rates ranging from 0.2 to 1 mV s^−1^ (Figure 4d). Clearly, all curves exhibit similar shapes with a small shift along with cycling. The degree of capacitive contribution can be analyzed by the relationship between the current (*i*) and the scan rate (*v*).

(2) $$i=av^{b}$$

Generally, the *b* value in Equation (2) can be obtained through the log(*i*) and log(*v*) plot, *b* = 0.5 corresponds to a diffusion‐controlled process, while *b* = 1.0 relates to a surface capacitive‐controlled process.^[^ ^36^ ^]^ The results of Figure 4e show that the *b* values of anodic (peak A) and cathodic peak (peak B) of SiO@NC‐NCNTs are 0.78 and 0.70, respectively, suggesting the capacitive‐guided mechanism for SiO@NC‐NCNTs. The capacitive contribution can be further determined via the relationship between current value *i*(*v*) at fixed voltage (V), capacity contribution (*k*_1_ *v*), and diffusion contribution (*k*_2_ *v* ^1/2^).

(3) $$i(V)=k_{1}v+k_{2}v^{1/2}$$

As observed from Figure 4f, the capacitive contribution is 63.04% of the total capacity at 1.0 mV s^−1^, and the proportion of capacitive contribution increases with increasing scanning rate. The results illustrate that the capacitive‐dominated behavior can store Li^+^ more effectively at a higher scan rate, which is in agreement with the superior rate capability of SiO@NC‐NCNTs.

**Figure 5:**
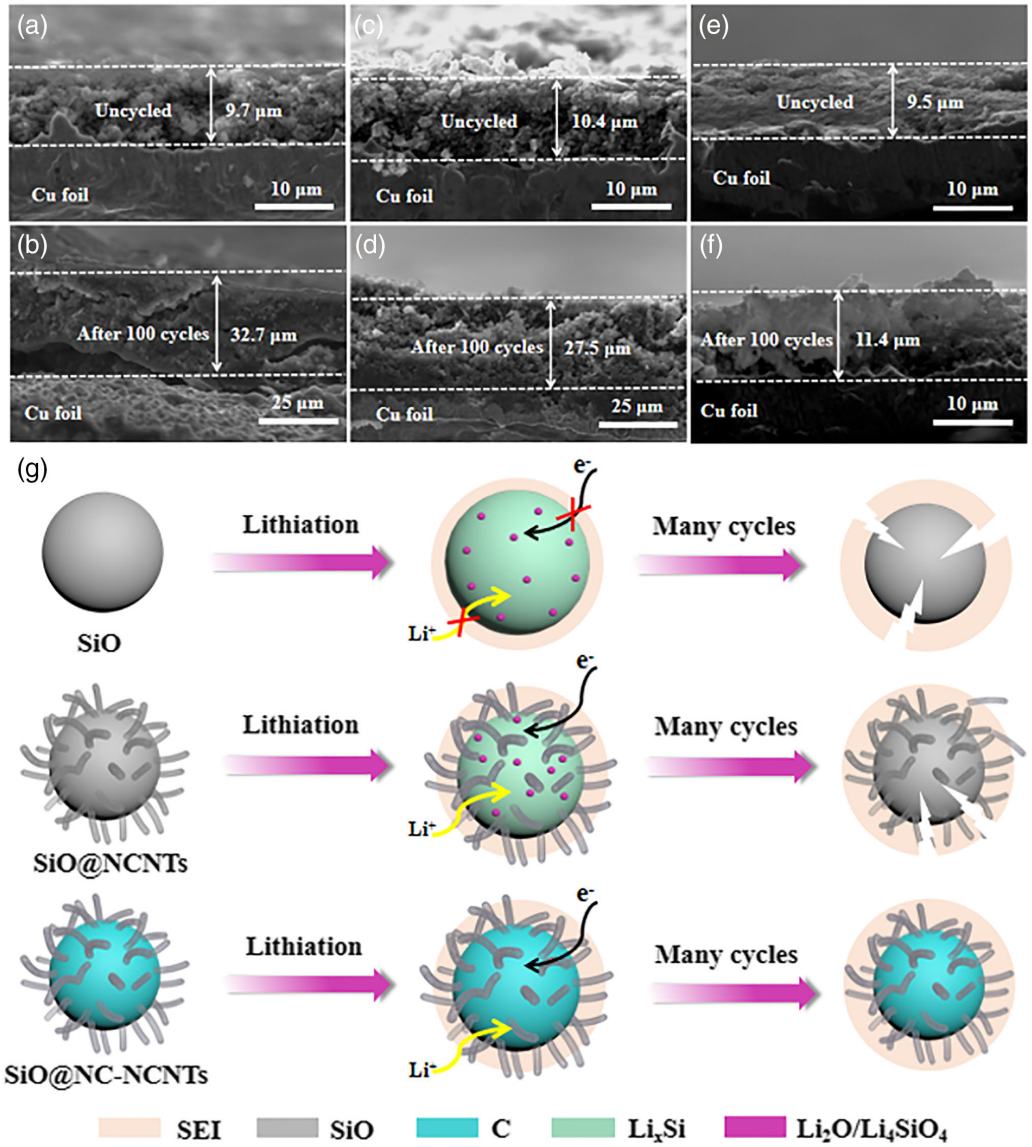
Cross‐sectional SEM images of the pristine SiO electrode a) before and b) after 100 cycles at 1.0 A g^−1^. Cross‐sectional SEM images of the SiO@NCNTs electrode c) before and d) after 100 cycles at 1.0 A g^−1^. Cross‐sectional SEM images of the SiO@NC‐NCNT electrode e) before and f) after 100 cycles at 1.0 A g^−1^. g) Schematic illustration of the structural changes of SiO, SiO@NCNT, and SiO@NC‐NCNT electrodes for LIBs after many cycles.

Then, the thickness changes of three electrodes after cycling to highlight the unique structural superiority of SiO@NC‐NCNTs were analyzed. Seen from the cross‐sectional SEM images (**Figure** **5**a,b), the thickness of the pristine SiO electrode dramatically increases from 9.7 to 32.7 μm after 100 cycles, and the cracking phenomenon can be obviously observed, which mainly result from the repeated large volume expansions during cycles, leading to structural degradation.

Figure 5g describes the possible morphological evolution of SiO, SiO@NCNTs, and SiO@NC‐NCNTs electrodes upon cycling. For pristine SiO, the low conductivity and large volume expansion lead to slow ion−electron transport and serious polarization of the electrode material.^[^ ^37^ ^]^ Although the introduction of NCNTs can shorten the ion diffusion path and improve the conductivity, falling off from the SiO surface due to weak bonding can happen. Fortunately, the problem can be solved through a smart design of the flexible middle NC layer. The strong combination of the middle NC layer and NCNTs can provide short transfer channels for Li ions and electrons and realize fast charge transfer kinetics. Accordingly, the buffering capability and cycling stability of SiO@NC‐NCNTs have been greatly improved.

**Figure 6:**
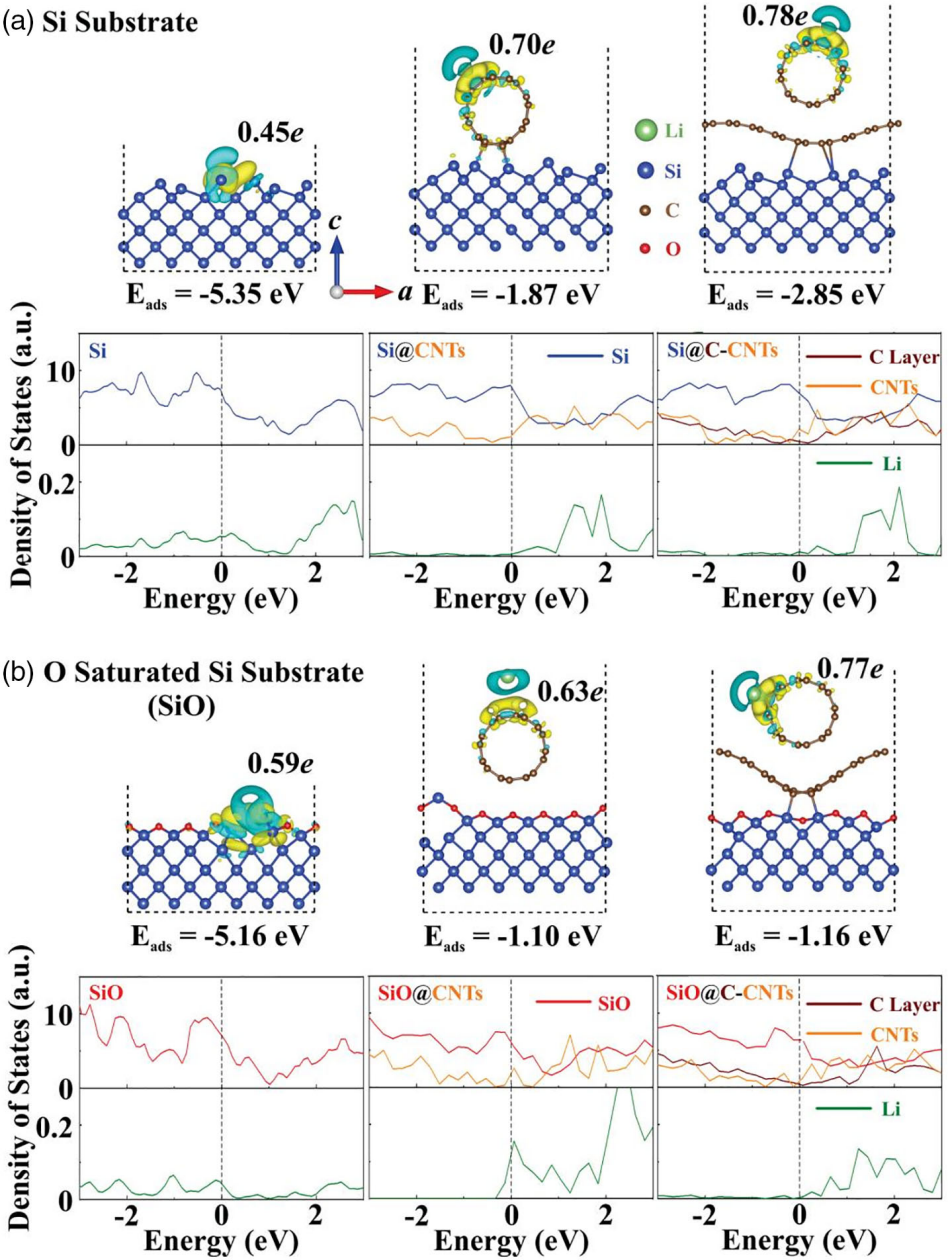
Charge density difference and the corresponding density of states for Li‐adsorbed a) Si, Si@CNTs, Si@C‐CNTs and b) SiO, SiO@CNTs, and SiO@C‐CNTs, respectively. The yellow and blue areas represent the electron accumulation and depletion, respectively. Fermi levels (dashed lines) are set to be zero.

On the basis of the experimental results, the first‐principle calculation was adopted to further reveal the underlying reason for the enhanced lithium storage performance of the ternary SiO@NC‐NCNT composite anode. In this case, the Si‐ or O‐saturated Si surfaces with and without CNTs are considered as the references. As displayed in **Figure** **6**, the electrons are mainly depleted around Li and accumulate around the surface Si or O or CNTs, indicating the strong electron transfer between Li and heterostructures. For Si or O exposed surface, Li atom is strongly bonded with surface atoms, leading to an extremely high adsorption energy (−5.35 and −5.16 eV for Si and O surface, respectively). Such high energy will hardly move Li atoms or release them from the surface, which may show an impact on the battery charging/discharging process. When loading CNTs onto the exposed Si or O surface, the adsorption energy will shift to a moderate range, reaching −1.87 and −1.10 eV for Si@CNTs and SiO@CNTs, respectively. By further adding the carbon layer, it will screen the interaction between CNTs and Si or O surface and slightly enhance the Li adsorption performance with the energy reaching up to −2.85 eV. Bader charge analysis shows that 0.78*e* and 0.77*e* electron transfer from Li to CNTs in Si@C‐CNTs and SiO@C‐CNTs, respectively, which are much larger than those in other cases. Therefore, the formation of crystal Si in the heterostructure will improve the Li adsorption performance. Then, the density of states for each substrate under Li adsorbed situations was examined. Due to the surface atomic distortion of Si, the Li adsorbed heterostructures possess the metallicity with the large electronic states of Si or SiO existing near the Fermi level. In addition, the electrons’ states of CNTs and carbon layer also demonstrate strong coupling with those of Si and SiO near the Fermi level, leading to the improved electronic conductivity. Therefore, the SiO@C‐CNTs heterostructure with balanced Li adsorption energies and improved electronic conductivity can be used as the promising material platform for LIB applications.

## 3 Conclusion

In conclusion, N‐doped CNTs are in situ grown onto PDA‐coated microsized SiO particles based on a facile metal catalytic pyrolysis approach. The strong coupling of 1D NCNTs with the flexible middle NC layer endows the SiO anode with excellent properties including a large surface area of 143.2 m^2^ g^−1^ and a highly stable 3D conductive network. With these structural and compositional advantages, the as‐obtained SiO@NC‐NCNTs electrode demonstrates a high reversible specific capacity of 1103.7 mA h g^−1^ at 0.2 A g^−1^ and a superior rate performance of 569 mA h g^−1^ at 5000 mA g^−1^. Compared with SiO@NCNTs and pristine SiO electrodes, the SiO@NC‐NCNTs electrode has only about 20% particle level expansion, as confirmed by the cross‐sectional SEM images. Importantly, the DFT theoretical analysis further confirms that the ternary SiO@NC‐NCNTs heterostructure can balance the adsorption and release of Li and improve conductivity. These results indicate that the flexible middle NC layer can alleviate large volume changes and enhance electrical contact of SiO@NC‐NCNTs, thus boosting the Li‐storage capability. The current study is expected to promote the commercialization of microsized SiO‐based anodes in high‐energy‐density LIB applications.

## 4 Experimental Section

4.1

4.1.1

##### Materials

All chemical reagents were used as received without further purification. The microsized SiO powder (2–3 μm), dopamine hydrochloride, FeCl_3_·6H_2_O, CoCl_2_·6H_2_O, melamine, ethanol, and nitric acid solution (HNO_3_) were all purchased from Sinopharm Chemical Reagent Co., Ltd. Distilled water was used in all the experiments.

##### Preparation of SiO@polydopamine Particles

The core−shell SiO@polydopamine particles (SiO@PDA) were first prepared as follows. Typically, 100 mg of commercial SiO powder with a diameter of 2–3 μm was dispersed in 200 mL of tris‐buffer solution (pH = 8.5) under ultrasonication for 20 min, and then 20 mg of dopamine hydrochloride and 18.4 mg of FeCl_3_·6H_2_O were added to the above solution and ultrasonically treated for 10 min to form a homogeneous suspension. After reacting at room temperature for 24 h under continuous stirring, the resulting precipitates were harvested by centrifugation and washed with deionized water and ethanol several times and dried at 60 °C in a vacuum oven overnight to get the core−shell‐structured Si@PDA particles.

##### Preparation of Microsized SiO@NC‐NCNTs Composite

The microsized SiO@NC‐NCNTs composite was synthesized via a facile catalytic pyrolysis technique, where melamine and CoCl_2_·6H_2_O were used as the carbon precursor and the catalyst, respectively. In an optimized synthesis, 200 mg of CoCl_2_·6H_2_O, 800 mg of melamine, 80 mg of Si@PDA synthesized earlier, and 8 mL of ethanol were homogeneously mixed. Then, the resulting powder was heated in a tube furnace at 800 °C for 3 h with a heating rate of 4 °C min^−1^ under N_2_ atmosphere to convert the PDA into N‐doped carbon layer (NC), which then in situ catalyzed the growth of N‐doped CNTs (NCNTs) onto SiO@PDA particles. After that, the obtained product was dispersed in 30 mL of HNO_3_ solution (6.5 m) and reacted at 60 °C for 12 h under stirring to remove the Co nanoparticles. After being cooled naturally, the microsized SiO@NC‐NCNT composite was collected by centrifugation and washed thoroughly by deionized water and dried at 60 °C overnight under vacuum. As the comparison, the SiO@NCNTs product without the middle NC layer was also prepared following the same procedure without PDA coating.

##### Material Characterizations

The powder XRD measurements were recorded on a Japan Rigakul D/max‐2550 instrument using Cu Kα radiation (*λ* = 0.154 nm). The morphology and composition of the samples were examined using SEM (ZEISS Gemini 300 and HITACHI SU‐1500), TEM (JEOL 200CX), and HRTEM (JEOL‐2000), accompanied by energy‐dispersive X‐ray spectroscopy (EDX, OXFORD Xplore). Raman spectra were obtained on the InVia confocal Raman microspectrometer. X‐ray photoelectron spectra (XPS) were recorded on the Thermo Scientific K‐Alpha XPS device with monochromatized Al Kα radiation. Thermogravimetric analysis (TGA) was conducted via the TA TGA Q50 instrument under flowing air atmosphere. The nitrogen adsorption/desorption isotherms were measured using a micromeritics ASAP 2010 analyzer.

##### Electrochemical Measurements

The electrochemical performances of the samples were evaluated by assembling CR2032‐type coin half cells in an argon‐filled glove box. Lithium metal was used as the counter electrode, and 1 m LiPF_6_ dissolved in ethylene carbonate/diethyl carbonate (1:1 by volume) with 5% fluoroethylene carbonate was employed as the electrolyte. The working electrodes were prepared by a doctor‐blade process, where the electrode slurry consisted of 80 wt% active materials. 10 wt% super‐P and 10 wt% sodium alginate were cast onto the copper foil and dried at 70 °C for 12 h in a vacuum oven. The mass loading of the active materials was 0.8–1.0 mg cm^−2^. A LAND‐CT2001A system was employed for the galvanostatic charge−discharge tests at different current densities at the voltages between 0.005 and 2.00 V, and the GITT analysis was also used. The CV and EIS were recorded with a CHI 660D electrochemical work station.

##### Computational Details

The first‐principles calculations were based on density functional theory (DFT) as implemented in the Vienna ab initio simulation package (VASP).^[^ ^38^, ^39^ ^]^ The functional for exchange correlation was described by the Perdew−Burke−Ernzerhof (PBE) functional within generalized gradient approximation (GGA).^[^ ^40^ ^]^ A more than 15 Å vacuum layer along the *z*‐direction was adopted to screen the interactions caused by structural periodicity. The first Brillouin zone was sampled by the 7 × 9 × 1 and the kinetic cutoff energy was set to be 400 eV. To simulate the slab structure and reduce the cost of calculations, the bottom two‐layer silicon atoms during the structural optimization were fixed. The force and energy for each atom were relaxed to be less than 0.001 eV Å^−1^ and 10^−6^ eV, respectively. DFT‐D3 correction was adopted to correct the van der Waals interactions. In the calculation, a surface of silicon crystal to model the amorphous silicon oxide and silicon was used due to the difficulties on DFT calculations. Then, to illustrate the influence of the existence of oxygen atoms, the surface silicon atoms with the oxygen ones (SiO) were replaced and their Li adsorption behaviors were investigated. In addition, the adsorption energy (*E*_ads_) was obtained by

(4) $$E_{\text{ads}}=E_{\text{Li‐Sub}}-E_{\text{Sub}}-E_{\text{Li}}$$

where *E*_Li‐sub_ and *E*_sub_ are the energies of the Si‐based substrates with and without Li adsorption, respectively, while *E*_Li_ represents the energy of the single Li atom.

## Conflict of Interest

The authors declare no conflict of interest.

## Supporting information

Supplementary Material

## Data Availability

Research data are not shared.
