# Supplementary material for: Adina Rubella‐Like Microsized SiO@N‐Doped Carbon Grafted with N‐Doped Carbon Nanotubes as Anodes for High‐Performance Lithium Storage
Source: Small Sci. 2022 Jan 12;2(4):2100105. doi: 10.1002/smsc.202100105 (PMC11935865; doi:10.1002/smsc.202100105)
Supplement: Supplementary file 1 — Supplementary Material [file SMSC-2-2100105-s001.pdf]

Supporting Information

Adina rubella-like micro-sized SiO@N-doped carbon grafted with N-doped CNTs as anodes for high-performance lithium storage

Weilan Xu,[a] Cheng Tang,[b] Na Huang,[a] Aijun Du,[b] Minghong Wu,[c] Jiujun Zhang,[d]* and Haijiao Zhang[a]*

[a]*Institute of Nanochemistry and Nanobiology, Shanghai University, Shanghai 200444, China*

[b]*School of Chemistry, Physics and Mechanical Engineering, Science and Engineering Faculty, Queensland University of Technology, Brisbane, QLD 4001, Australia*

[c]*School of Environmental and Chemical Engineering, Shanghai University, Shanghai 200444, China*

[d]*Institute for Sustainable Energy, College of Sciences, Shanghai University, Shanghai, 200444, China*

*Corresponding author

E-mail: hjzhang128@shu.edu.cn, jiujun.zhang@i.shu.edu.cn

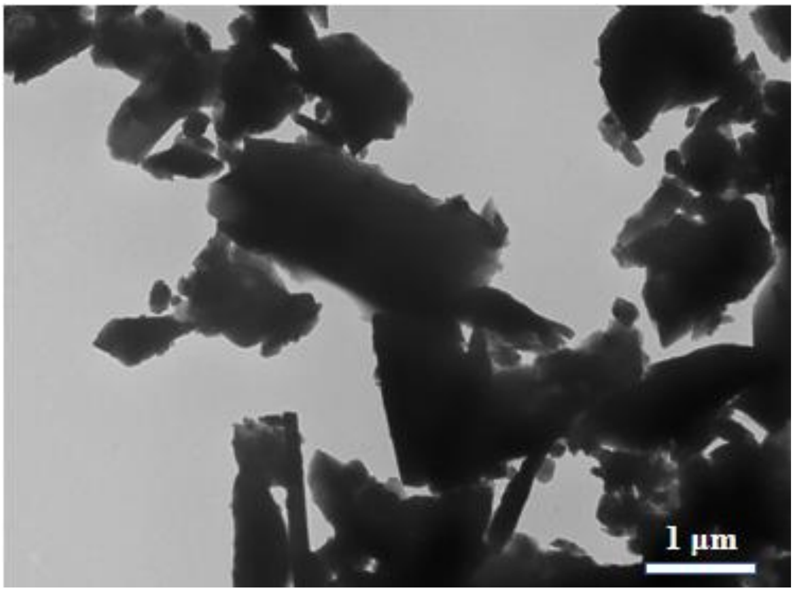

Figure S1. TEM image of pristine SiO particles.

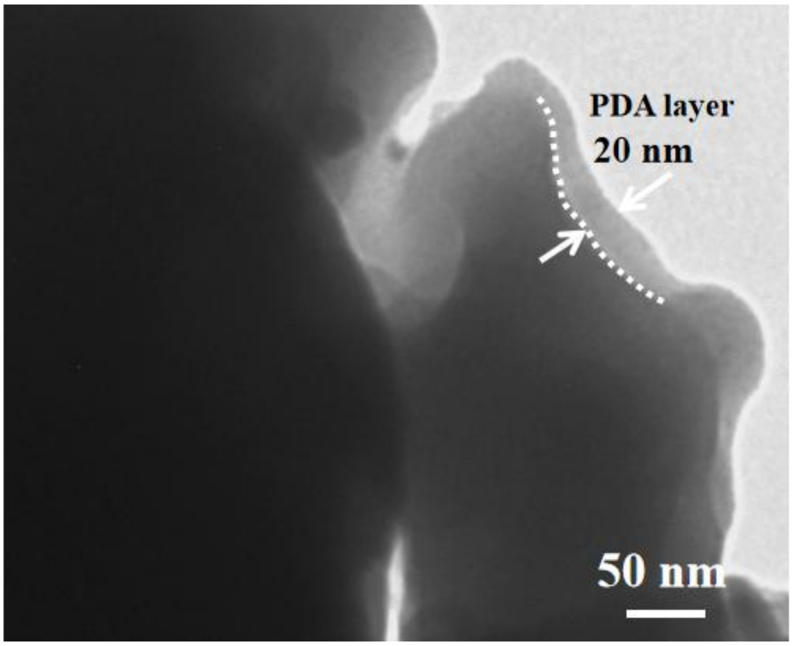

Figure S2. TEM image of SiO@PDA.

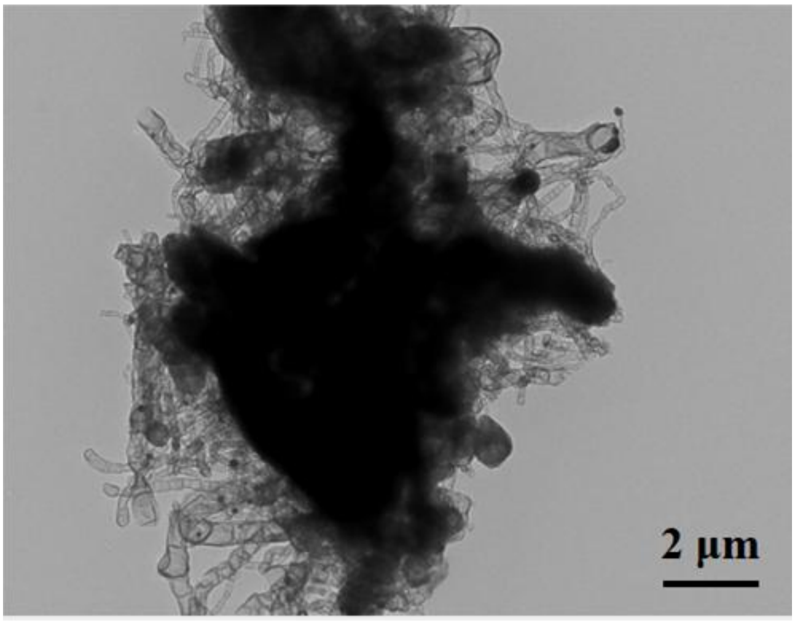

Figure S3. TEM image of SiO@NCNTs.

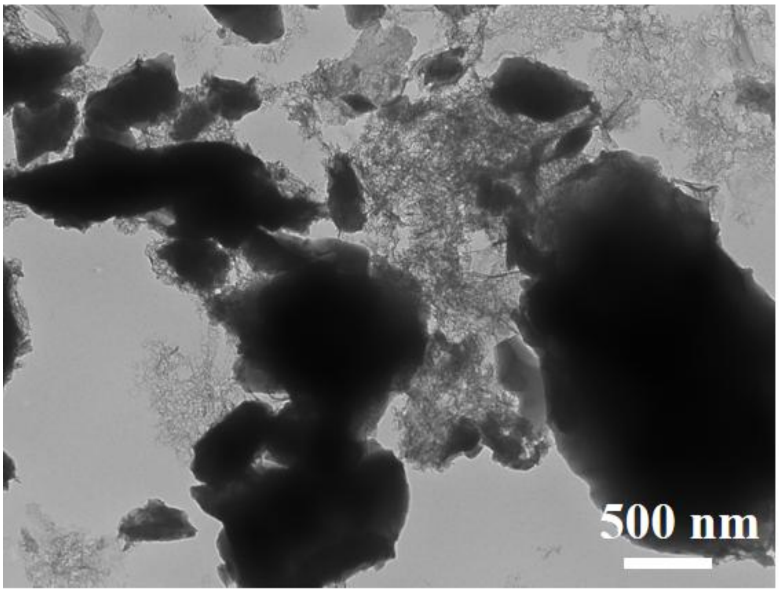

Figure S4. TEM image of the sample synthesized without Co catalyst.

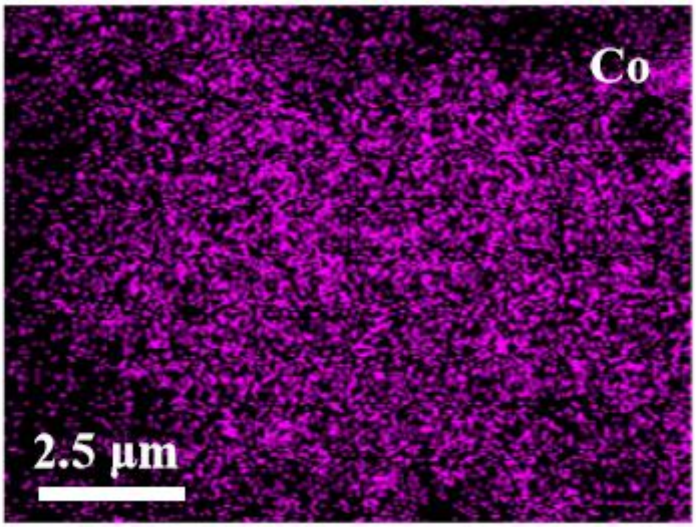

Figure S5. The elemental mapping of Co.

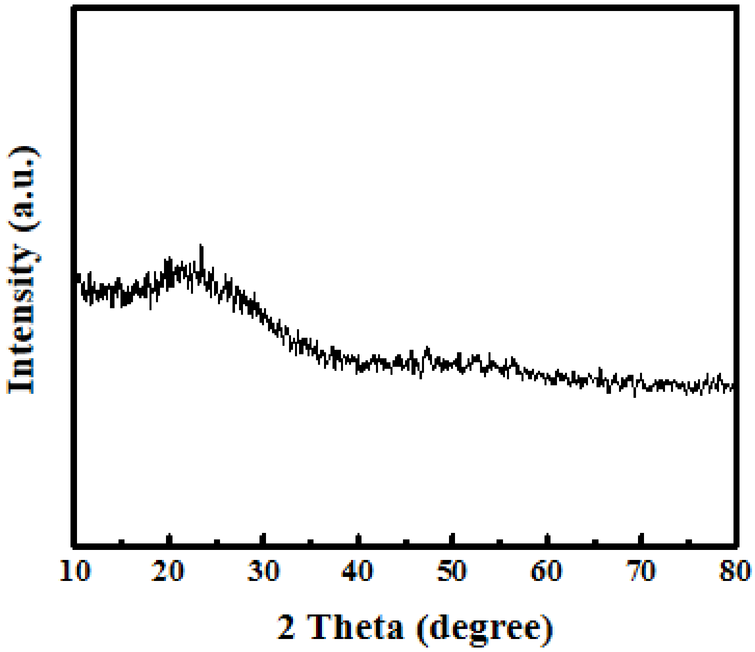

Figure S6. XRD pattern of pristine SiO particles.

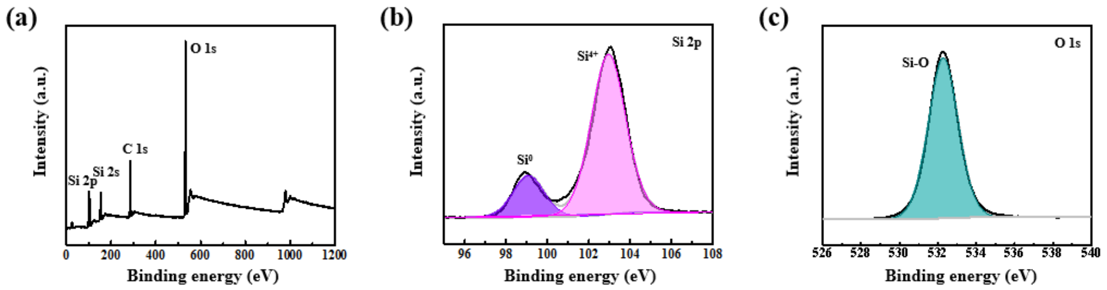

Figure S7. a) XPS survey spectrum and high-resolution XPS spectra of b) Si 2p, and c) O 1s of SiO.

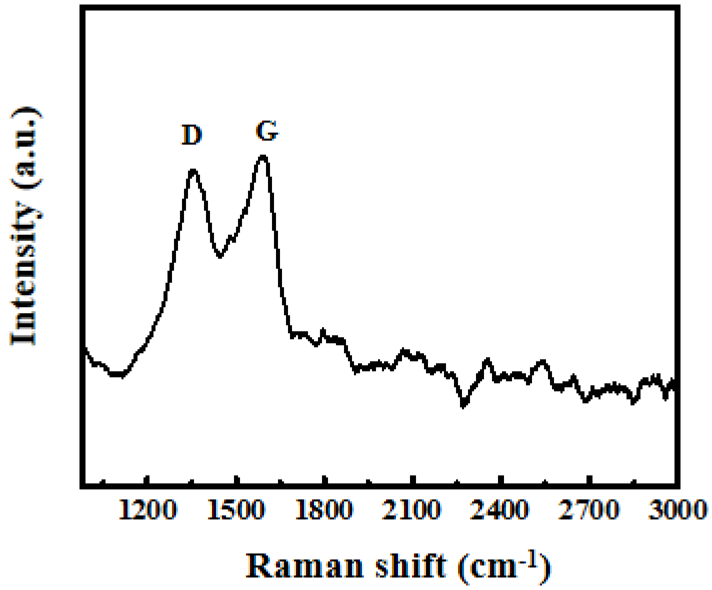

Figure S8. Raman spectra of the sample synthesized without Co catalyst.

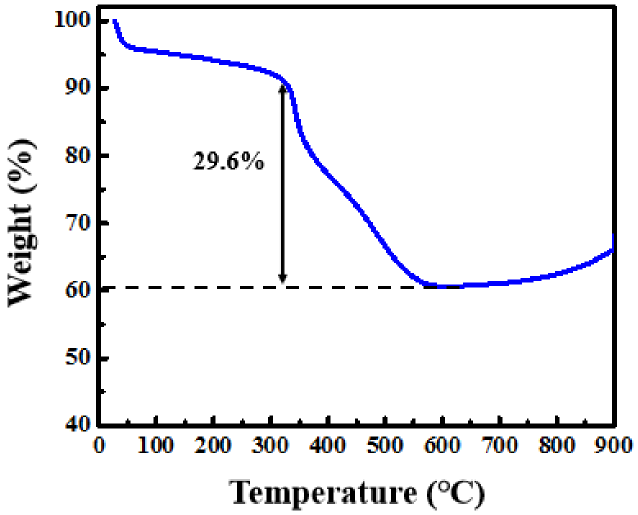

Figure S9. TGA curve of SiO@NCNTs.

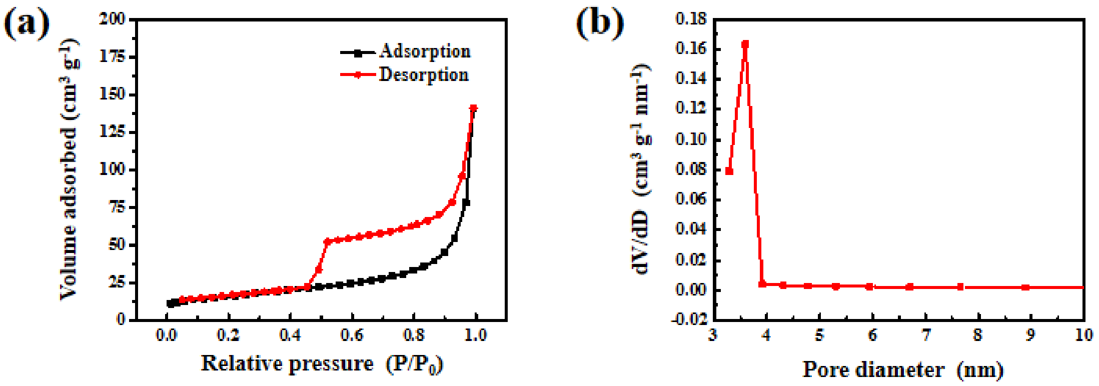

Figure S10. (a) N_2 adsorption-desorption isotherm, and (b) corresponding pore size distribution curve of SiO@NCNTs.

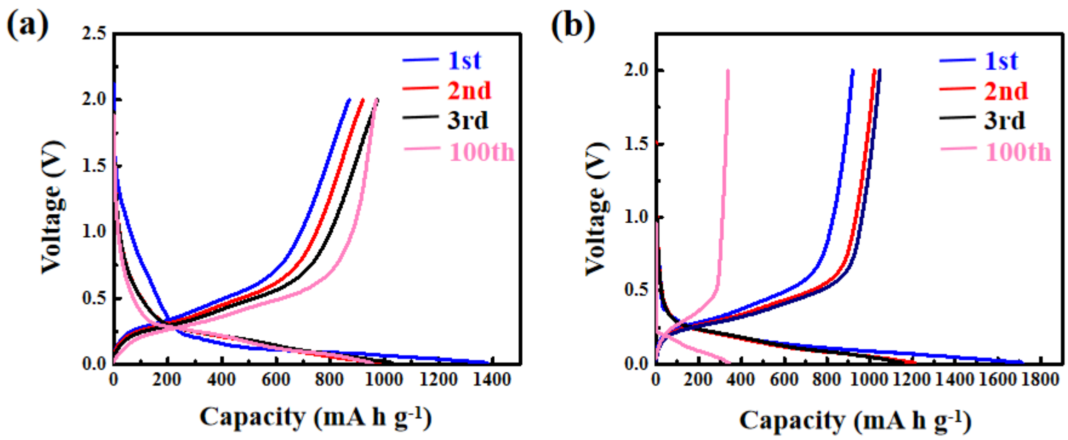

Figure S11. Charge-discharge profiles of (a) SiO@NCNTs, and (b) pristine SiO electrodes.

Table S1. Comparison of electrochemical performances of the SiO@NC-NCNTs electrode with other Si-based anodes for lithium-ion batteries.

Electrode materials	Current density (mA g^{-1})	Gravimetric capacity (mAh g^{-1})	Cycle number	Ref.
**SiO@NC-NCNTs**	**200**	**1103**	**200**	**This work**
Si /red P/graphite	200	883.4	200	[1]
C-SiOx/C	750	836	500	[2]
Porous C@Si	100	934	100	[3]
SiOx@NC	200	774	100	[4]
C@Si	400	738	120	[5]
Ti_3C_2@Si/SiOx@TiO_2	100	939	100	[6]
C@SiO	100	300	500	[7]
SiOx@C	100	1115.8	200	[8]
C@void@Si	200	854.1	200	[9]
SiOx-TiO_2/RGO	100	730	200	[10]

References

[1] S. Huang, L. Z. Cheong, D. Wang, C. Shen, Nanostructured phosphorus doped silicon/graphite composite as anode for high-performance lithium-ion batteries, ACS Appl. Mater. Interfaces 9 (2017) 23672-23678.

[2] G. Li, L.-B. Huang, M.-Y. Yan, J.-Y. Li, K.-C. Jiang, Y.-X. Yin, S. Xin, Q. Xu, Y.-G. Guo, An integral interface with dynamically stable evolution on micron-sized SiOx particle anode, Nano Energy 74 (2020) 104890.

[3] P. Guan, J. Li, T. Lu, T. Guan, Z. Ma, Z. Peng, X. Zhu, L. Zhang, Facile and scalable approach to fabricate granadilla-like porous-structured silicon-based anode for lithium ion batteries, ACS Appl. Mater. Interfaces 10 (2018) 34283-34290.

[4] G. Hu, K. Zhong, R. Yu, Z. Liu, Y. Zhang, J. Wu, L. Zhou, L. Mai, Enveloping SiOx in N-doped carbon for durable lithium storage via an eco-friendly solvent-free approach, J. Mater. Chem. A 8 (2020) 13285-13291.

[5] Z. Li, Z. Li, W. Zhong, C. Li, L. Li, H. Zhang, Facile synthesis of ultrasmall Si particles embedded in carbon framework using Si-carbon integration strategy with superior lithium ion storage performance, Chem. Eng. J. 319 (2017) 1-8.

[6] M. Jiang, F. Zhang, G. Zhu, Y. Ma, W. Luo, T. Zhou, J. Yang, Interface-amorphized Ti_3C_2@Si/SiOx@TiO_2 anodes with sandwiched structures and stable lithium storage, ACS Appl. Mater. Interfaces 12 (2020) 24796-24805.

[7] L. Feng, X. Han, X. Su, B. Pang, Y. Luo, F. Hu, M. Zhou, K. Tao, Y. Xia, Metal-organic frameworks derived porous carbon coated SiO composite as superior anode material for lithium ion batteries, J. Alloys Compd. 765 (2018) 512-519.

[8] Z. Li, H. Zhao, J. Wang, T. Zhang, B. Fu, Z. Zhang, X. Tao, Rational structure design to realize high-performance SiOx@C anode material for lithium ion batteries, Nano Res. 13 (2020) 527-532.

[9] H. Mi, X. Yang, Y. Li, P. Zhang, L. Sun, A self-sacrifice template strategy to fabricate yolk-shell structured silicon@void@carbon composites for high-performance lithium-ion batteries, Chem. Eng. J. 351 (2018) 103-109.

[10] Y. Jiang, S. Liu, Y. Ding, J. Jiang, W. Li, S. Huang, Z. Chen, B. Zhao, J. Zhang, Modification based on primary particle level to improve the electrochemical performance of SiO-based anode materials, J. Power Sources 467 (2020) 228301.
